# Supplementary material for: High-dose vitamin D versus placebo to prevent complications in COVID-19 patients: Multicentre randomized controlled clinical trial
Source: PLoS One. 2022 May 27;17(5):e0267918. doi: 10.1371/journal.pone.0267918 (PMC9140264; doi:10.1371/journal.pone.0267918)
Supplement: S1 File — (PDF) [file pone.0267918.s002.pdf]

Controlled trial with high-dose vitamin D versus placebo to prevent progressive complications in patients infected with COVID-19.

Short name: Colec**A**lcife**R**ol para m**E**jorar la evolución de pacientes con COVI**D**-19 (**CARED**)

Reseachers: Walter Manucha[1], Leon Ferder[2], Felipe Inserra[2], Javier Mariani[3,4], Laura Antonietti[3,4] ,Carlos Tajer[3,4] .

Affiliation:

[1] Instituto de Medicina y Biología Experimental de Cuyo (IMBECU), CCT-Mendoza, CONICET, Facultad de Ciencias Médicas, Universidad Nacional de Cuyo, Av. Ruíz Leal, s/n Parque Gral. San Martín (5500), Mendoza, Argentina.

[2] Universidad Maimónides, Hidalgo 775 (C1405), Ciudad Autónoma de Buenos Aires, Argentina.

[3] Hospital de Alta Complejidad El Cruce Néstor Kirchner, Av. Calchaquí 5401 (C1888), Florencio Varela, Buenos Aires, Argentina.

[4] Universidad Nacional Arturo Jauretche, Av. Calchaquí 6200 (C1888), Florencio Varela, Buenos Aires, Argentina.

1. Introduction

A new coronavirus (SARS-CoV-2) has generated a pandemic with a large number of individuals infected and dying globally from the respiratory complications of the virus (1).

SARS-CoV-2 causes COVID-19 disease by entering alveolar epithelial cells through its affinity for angiotensin-converting enzyme ACE-2 receptors (2). The reduction of the induced ACE-2 enzyme activity is a pathogenic mechanism of coronaviruses at pulmonary level, by exacerbation of the local activity of angiotensin II that induces an active inflammation (3). Earlier reports of patients infected by COVID-19, the highest risk of complications and mortality was associated with advanced age, hypertension, diabetes, pulmonary and cardiac diseases. These clinical conditions have exaltation of the renin-angiotensin system (RAS) (4-8) with low vitamin D status (9). Coincidentally, pulmonary complications and deaths in viral infections have an inverse relationship with serum vitamin D levels (10-14). Multiple studies show that vitamin D would have an antagonistic effect on RAS at the tissue level, reducing the inflammatory response (9, 15-23). In this context treatment to increase vitamin D levels could be beneficial.

Our assumption is based on our own and other authors' evidence, which provides epidemiological and clinical information on the modulation of the inflammatory pathway in severe viral infections by vitamin D, with the particularity of the interaction of COVID-19 on the ACE2 enzyme and the modulation exerted by vitamin D on it (Figure 1 and Figure 2). Therefore, supplementing vitamin D could provide protection against COVID-19, both in the prevention and in the development and/or progression of the disease. Given this background and the magnitude of the pandemic, it is essential to explore the role of vitamin D in preventing and reducing the complications of COVID-19 infection (24, 25). Administration of a single high dose of vitamin D has been shown to increase blood levels of 25 (OH) vitamin D to sufficient levels and these levels remain elevated for more than one month; these therapeutic effects are achieved with minimal incidence of mild adverse events (26). The use of a single 500,000 IU dose of cholecalciferol is justified by the relevance of achieving adequate serum vitamin D levels within a short period of time from the onset of SARS-CoV-2 infection. Moreover, considering the dynamics of COVID-19 and

its potential respiratory complications, a single dose would allow covering the entire period of increased risk with an excellent safety profile. Although there is some debate about possible toxic effects, these have been reported mainly with chronic administration of vitamin D, with insufficient reports of significant toxicity with the use of a high single dose, while there is vast scientific evidence of effectiveness, devoid of toxic effects so far.

The aim of the study is to evaluate whether high-dose vitamin D administration is an effective intervention to decrease the rate of respiratory and systemic complications in patients with confirmed COVID-19 infection.

Figure 1: Signaling pathways in the potential therapeutic effects of vitamin D in COVID-19 disease.

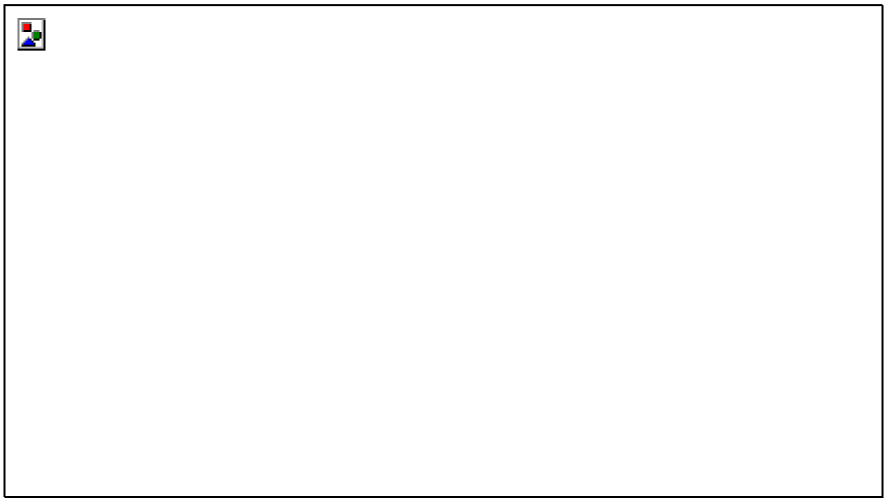

Figure 2: Interaction between the renin-angiotensin system and vitamin D (potential therapeutic implications).

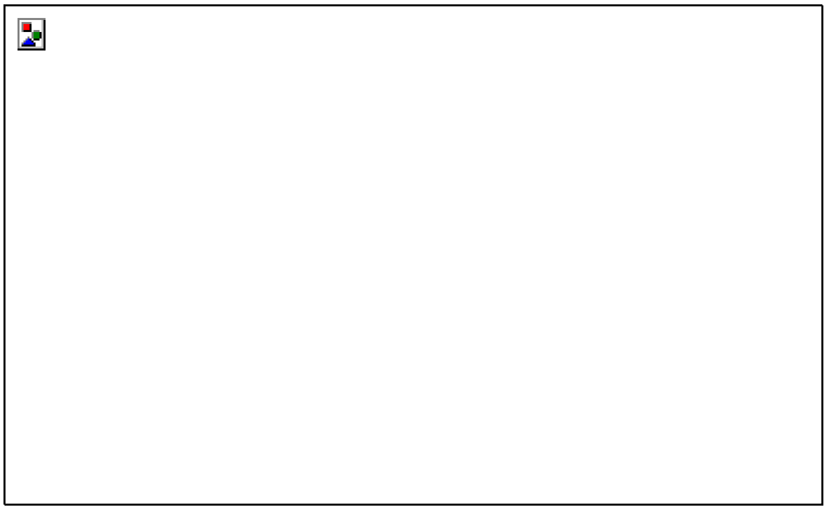

2. Material and Method

a. Design

A randomized, multicenter, double-blind, parallel-group, sequential, placebo-controlled, multicenter clinical trial was designed to evaluate the effects of high-dose vitamin D on the outcome of patients with a confirmed diagnosis of COVID-19 infection.

b. Participants

Individuals who meet the following inclusion criteria and none of the exclusion criteria will be considered for the study:

- **Inclusion criteria**
  - Laboratory confirmation of SARS-CoV-2 virus by PCR or standard methodology used by the research center;
  - Hospitalization in a health care facility;

- Planned hospitalization in the same facility for at least 24 hours;
- Arterial oxygen saturation greater than 90% when breathing room air;
- Age ≥45 years and/or at least one criterion associated with an increased risk of progressive complications according to COVID-19: Arterial hypertension, History of diabetes I or II, History of asthma/COPD, Cardiovascular history (history of myocardial infarction, coronary angioplasty, CABG or valve replacement surgery), Obesity (body mass index ≥30);
- Willingness to sign informed consent for the study.

- **Exclusion criteria**
  - Age under 18 years old;
  - Having been hospitalized for 72 hours in any health care facility;
  - Women of childbearing age (if less than one year has elapsed since the date of the last menstrual period at the time of the evaluation);
  - Requirement of high-flow oxygen therapy, non-invasive ventilation (NIV) or invasive ventilation (mechanical ventilation);
  - Chronic renal insufficiency on dialysis or known hepatic insufficiency;
  - Oral intolerance;
  - Chronic pharmacological treatment with vitamin D;
  - History of:
    - Treatment with any anticonvulsant medication;
    - Sarcoidosis;
    - Malabsorption síndromes;
    - Known hypercalcemia o Calcemia greater than 10.5 mg/dL.
  - Systemic disease considered terminal (life expectancy less than six months);
  - Known allergy to study medication;
  - Any condition that precludes giving informed consent.

c. Intervention

A single dose of 500,000 IU of Vitamin D3 (cholecalciferol) or placebo will be administered. The study medication will consist of 5 soft capsules containing 100,000 IU of Vitamin D or

placebo each. With this dose of vitamin D3, sufficient plasma 25 (OH) vitamin D levels have been observed as early as 24 h and persistence within the range >29 ng/ml beyond one month of administration (26).

d. Outcomes

The protocol has a sequential design, in which a first objective related to the evolution of oxygenation will be evaluated. If a favorable result is obtained in the interim analysis, patient recruitment will be expanded and additional objectives will be evaluated.

In the second stage of the study, the first secondary endpoint of the first stage will become a primary combined efficacy endpoint.

- **First Stage**
  - **Primary Outcome**

Change in respiratory SOFA score between admission and the worst recorded respiratory SOFA (arterial O_2 saturation measured by pulse oximeter will be used to estimate it) (28).

- **Secondary Outcomes**

Secondary objectives of the study will be analyzed until discharge from hospitalization.

1) Change in O_2 saturation between admission and the worst O_2 saturation recorded.

2) Desaturation: O_2 saturation less than or equal to 90% breathing room air at any time during hospitalization, measured by pulse oximeter (SpO_2).

3) Combined end point: requirement for assistance with high-flow oxygen therapy (>40% inspired fraction O_2), indication for noninvasive or invasive ventilation.

4) Change in quick SOFA score from admission to worst score recorded during hospitalization.

5) Combined incidence of stroke, acute renal failure, myocardial infarction and/or pulmonary thromboembolism.

6) Admission to the Intensive Care Unit for clinical needs.

7) Requirement of invasive mechanical ventilation.

8) Length of hospital stay.

9) Length of intensive care stay.

10) In-hospital death.

- **Second Stage**

If favorable results are obtained at the end of the first stage, and by decision of the trial's Executive Committee, the second stage of the protocol will be continued.

- **Primary Outcome**

Combined event: requirement for assistance with high-flow oxygen therapy (>40% inspired fraction O_2), indication for noninvasive or invasive ventilation.

- **Secondary Outcomes**

1) Admission to the Intensive Care Unit for clinical needs.
2) Requirement of invasive mechanical ventilation.
3) Length of hospital stay.
4) Length of intensive care stay.
5) In-hospital death.

e. Pathophysiological substudy:

For the purpose of pathophysiological analysis, in selected centers in the City of Buenos Aires and the Province of Buenos Aires, a subgroup of patients enrolled in the study will have a blood sample taken at admission and another blood sample taken between days +3 to +7, or the day of hospital discharge (whichever occurs first). An aliquot of the samples will be used to assess parameters of inflammatory activity, renin angiotensin system and plasma OH vitamin D levels reached.

The remaining samples will be stored for possible future measurements of scientific interest. All samples will be stored in a coded manner in order to guarantee the confidentiality of patient data. Prior to sample collection, each patient who has agreed to participate will be invited to participate in the pathophysiological substudy and will be

asked to provide specific consent for the substudy. (See appendix: Informed consent form for the physiopathological substudy).

f. Collection of information

All information will be entered into an electronic case report form (eCRF) with encrypted and anonymized data. The database
will be constructed ad hoc for the clinical trial and will comply with current legislation on personal data protection (Law 25.326).
All information regarding the study subjects will be stored in password-protected computer files or in locked cabinets to which only authorized study personnel will have access. Biological samples, charts and files will be identified by a unique identification number. Data from questionnaires and clinical records will be entered into the database. The database will be backed up weekly and stored in duplicate on a server to which only the study's Executive Committee has access.
Biological samples will be stored for future research for a maximum period of 15 years, and may be analyzed only for scientific purposes. The specific informed consent of the sub-study incorporates this item, making explicit the right of the participants to communicate at any time with the principal investigator so that they may be destroyed and no longer used for eventual analysis, if they so wish.

g. Follow-up

Patient follow-up will be limited to hospitalization. Pathophysiological end points (O_2 saturation) will be recorded until day 7, death or discharge, whichever occurs first. Clinical end points will be recorded until death, discharge or one month, whichever occurs first.

h. Procedures of the study

The principal investigator of each center or other investigators designated by the principal investigator will evaluate the inclusion/exclusion criteria for potential study participants.
If they meet the inclusion criteria and none of the exclusion criteria, they will be invited to participate in the trial. After obtaining informed consent (see Appendix), the center investigator will collect and record in the CRF the patient's baseline information at the time of inclusion: axillary temperature, blood pressure (BP), heart rate (HR) and respiratory rate

(RR), O_2 saturation (SpO_2), inspired fraction of O_2 (FiO_2) and whether the patient is receiving supplemental O_2. Data regarding COVID-19 symptoms and relevant clinical history, including the patient's usual treatment, serum calcium level and other clinical laboratory test data - if routine tests have been performed - should also be recorded.

For the measurement of baseline SpO_2, the patient should be breathing room air for at least 5 minutes until steady-state pulse oximetry is achieved (29).

Electronic randomization of the patient will then be performed, through the eCRF. The investigator will record -both in the eCRF and in the clinical history- the treatment group and the assigned container number, will administer the study medication and will witness its intake.

On a daily basis the research team members will record: Axillary Tº, vital signs (BP, HR, FR), the minimum SpO_2 by pulse oximetry and the inspired fraction of O_2 at the time of measurement (FiO_2). These data will be recorded for the first seven days, until death or discharge, whichever occurs first. The saturation breathing room air, both at admission and at each evaluation, will be performed after 5 minutes of suspending the oxygen supply in case the patient is receiving some type of oxygen supply. In case it is not possible to suspend O_2 supply, saturation will be evaluated with the current O_2 supply and a saturation less than 90% will be assigned for the corresponding end point.

In addition, during follow-up, the following will be assessed and recorded in the eCRF: data on therapies indicated for COVID-19 management (not investigational treatments), clinical events (FiO_2 requirement >40%, need for NIV or invasive mechanical ventilation, transfer to intensive care unit – ICU-, Myocardial infarction, Stroke and Pulmonary thromboembolism) corresponding to clinical end points and serious adverse events up to 30 days, discharge or death, whichever occurs first. All event information will be entered into the electronic case report form (eCRF) to be designed for the study. Figure 3 shows the procedural outline of the study.

Considering that this is an independent, pragmatic study that does not involve extraordinary health care procedures (to avoid overloading the health teams in the context of the pandemic, according to the recommendations of the National Ministry of Health), in which the data to be collected and recorded will be obtained from routine care practice in patients with COVID-19, all measurements of vital signs and O_2 saturation as well as serum calcium will be carried out with the standard equipment used by the institution,

which are those accepted and provided by the jurisdictional authorities for routine clinical practice. Also, participation in the protocol does not interfere with or preclude the administration of any supportive therapy that is carried out by standard practice in the centers.

All inconsistencies will be recorded and monitored by the study coordinating center and queries will be generated and escalated to each principal investigator for resolution.

All clinical events and serious adverse events, in addition to being reported to the coordinating center, should be reported to the local Ethics Committee according to current regulations.

Figure 1: Study flow.

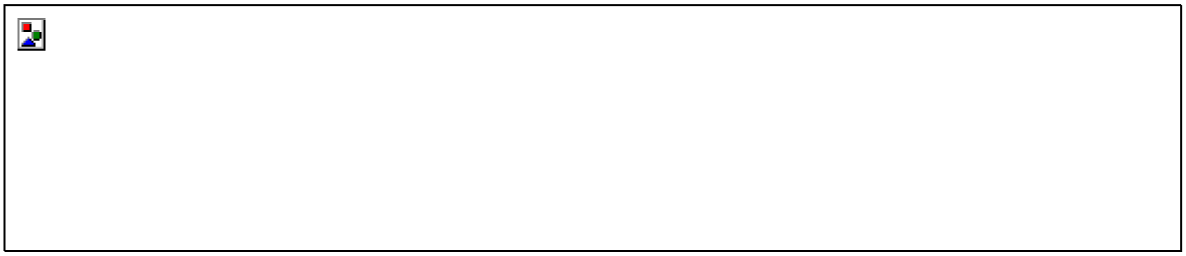

i. Double blind

Vitamin D will be available in soft capsules of 100,000 IU and the placebo will be a capsule identical in its organoleptic characteristics (shape, appearance, color, taste, smell and texture). All study medication will be packaged at the Maimonides University Pharmacy laboratory in bottles labeled with letter and package number. All bottles will be identical. The randomization lists will be stored at the coordinating center and the opening of the blind will only be accepted at the request of the principal investigator in cases of serious events for which knowledge of the treatment could modify clinical behavior.

The investigational products - active principle and placebo - will be elaborated, handled and stored according to Good Manufacturing Practices (GMP) in accordance with the provisions of ANMAT Provision 3287/18. The active principle will be obtained from a commercial form and repackaged to ensure blinding, while the placebo will be elaborated for the purposes of the protocol in a laboratory that complies with the requirements of the aforementioned regulation and repackaged at the Central Pharmacy of the University.

j. Research sites

The Executive Committee of the study will identify potential participating centers. After identification, contact will be established by e-mail and/or telephone with the centers' representatives to determine their interest in participating. In case of an affirmative response, a feasibility form will be sent to them to determine the center's possibilities of participating.

The criteria to be used are the willingness to participate, the availability of human resources to be assigned to the study and the number of patients that each center could potentially include.

If a center is selected, the protocol will be presented to the research team and then sent for approval by the institutional Research Ethics Committee. After approval, the study medication will be sent and training on study procedures will be accomplished at the center. Training visits at the centers will be conducted remotely in cases where social distance cannot be guaranteed.

Once the centers have the approval of their institutional IRB, the list of centers participating in the CARED Multicenter Network and their Principal Investigators will be compiled and informed to all IRBs and institutional authorities.

k. Randomization

A computer-generated, stratified, permuted-block, 1:1 computer-generated randomization scheme will be used.

The stratified randomization scheme will ensure the balanced distribution of relevant prognostic variables, and will be by two variables:

- Age: equal to or younger than 60 years or older than 60 years.
- Diabetes: yes or no.

Medication will be prepared in permuted blocks, which will be sent to each center, so as to ensure a balanced distribution of treatments in the strata at each of the study sites.

Treatment allocation will be done electronically using an internet-based interactive response system (IWRS).

l. Sample size considerations

For the first quantitative objective based on respiratory SOFA scores, 168 patients would be needed, 84 per group. Corrected for possible losses due to lack of compliance with the protocol, a total of 200 patients are estimated.

An interim analysis is planned when the 200 patients are reached in order to evaluate, if the results are positive, the expansion of the sample for clinical objectives.

In a stepwise fashion, if a positive result is obtained for the initial primary objective, the sample will be increased for the objective with the greatest clinical impact, which is to avoid oxygen therapy and progress to invasive ventilation (Figure 3). On the expectation of a need for inpatient oxygen therapy of 30% and a relative risk of 0.75, i.e., a 25% reduction, with an alpha error of 0.05 and a power of 80%, a total of 1,072 patients would be required, 536 per group. Adjusted for losses, the sample required is estimated at 1,265 patients.

Figure 3: Stages of the study.

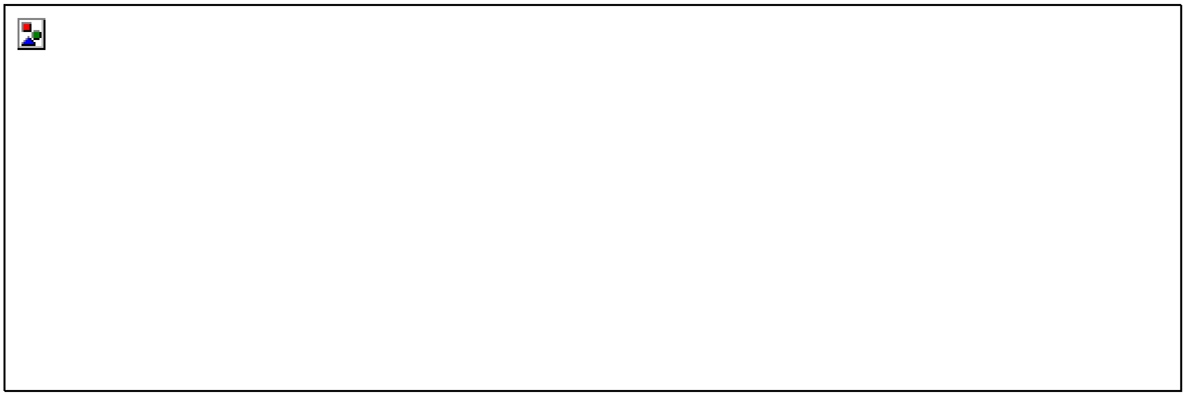

m. Screening and Recruitment

The number of patients needed to evaluate the results of the first stage is expected to be reached 45 days after the activation of the first center. For this purpose, at least 10 centers with cases of patients with COVID-19 in the period prior to the start of the study will be included in this stage. Thus, each center should include an average of 20 patients in this

time period. In case of a lower than expected recruitment rate, more centers will be included to increase it. In total, it is planned to include at least 15 centers in the study. This number could vary according to the results of the first stage and the recruitment rate in the study.

To achieve the objective of obtaining data 60 days (and reaching 200 participants 45 days) after the start of the study, 4.4 patients per day (about 1 patient every 2 days in each center) should be included, which we estimate to be feasible.

n. Statistical analysis

The description of quantitative data will be expressed as mean and standard deviation when normally distributed, or median and interquartile ranges for non-Gaussian data or scores. Qualitative data will be expressed as absolute and relative frequencies. Comparison of differences in SOFA scores between groups will be performed using the Mann-Whitney U test. The incidence of binary endpoints will be expressed as relative risks with their 95% confidence intervals and differences between groups will be evaluated with the Chi2 test or Fisher's Exact test, as appropriate. The numbers needed to treat and the absolute risk change will be derived from the result. The final expression of the results will be presented by subgroups with interaction test: age groups, diabetes, hypertension, sex, COPD, cardiovascular history, body mass index, place of residence (slums vs other locations), asthma and smoking. For all calculations, a significant p value <0.05 was considered significant. The analysis of the initial and final end point will be conducted following the intention-to-treat principle.

o. Interim analysis

An interim analysis will be performed after the incorporation of the first 200 patients. This analysis will aim to assess the effects of treatment on the first stage endpoints. The first stage is designed to have a power of 80% to detect a difference of at least one point in the respiratory SOFA. If a difference of this magnitude or greater is detected, proceed to the second stage of the study. In case of differences between 1 and 0.3 points, the Executive Committee of the study will discuss with the Data Safety and Monitoring Committee the strategy to be followed. This discussion will take into account the magnitude of the

difference, the effects on other endpoints, the level of significance and the eventual power of the study to detect smaller intervention effects than initially planned. The pathophysiological sub-analysis will be performed independently of the results of the first stage.

p. Safety of the study intervention

There is vast scientific evidence on the use of a single high dose of vitamin D, devoid of toxic effects. Reports of vitamin D intoxications from excess vitamin D come from chronic use mostly by megadoses (30).

A meta-analysis systematically reviewed the 30 clinical trials as of 2014 that used high single doses of vitamin D up to 600,000 IU. None of the trials reported adverse effects outside of digestive intolerance in a few cases. They observed mild hypercalciuria but in no trial increased incidence of kidney stones, significant hypercalcemia, or renal damage (31).

The controlled trial conducted by the US National Institute of Health, subsequent to this meta-analysis, confirmed the same findings, i.e., the absence of significant adverse effects. They evaluated 1078 patients with sepsis, who were given a vitamin D dose of 540,000 IU versus placebo. They observed no differences in the incidence of hypercalcemia, renal failure or kidney stones (there were only three in the placebo group and none in the Vitamin D group) (32).

In our protocol, with a single load of 500,000 IU, and based on the demonstrated evidence, although we would significantly raise circulating vitamin D levels - central objective - we would be far from the levels with evidence of toxicity, similar to all the trials that used high single doses, as we have documented in the summarized bibliography.

Adverse event monitoring and reporting will be performed during the study, in accordance with current regulations and as requested by the intervening institutional Ethics Committee. The safety profile of the dose and schedule (single dose) of the study is highly safe, with mild digestive symptoms (dyspepsia, nausea) having been reported. In addition, mild hypercalcemia, mild hypercalciuria and increased urinary magnesium were reported. All the reported alterations were laboratory manifestations, without clinical symptoms (26,29,33,34).

The protocol will also have an insurance policy to cover possible claims.

q. Ethical issues

Given that this is a Phase IV clinical trial involving the use of an active ingredient approved by the National Agency of Medicines, Food and Technologies (ANMAT) considering that the proposed dose is higher than the usual use and in the context of a health emergency, it corresponds to a higher than minimal risk research in accordance with the provisions of Decree 3385/09 of the Province of Buenos Aires and Resolution 2033/19 of the Ministry of Health of the Autonomous City of Buenos Aires.

Likewise, the research corresponds to a study without commercial interest, led by an investigator belonging to an academic institution and a responsible team endorsed by academic institutions, in accordance with the provisions of the technical opinion 001/2016 of the Joint Commission for Health Research of the Province of Buenos Aires (35). Following the ethical guidelines established by the WHO (36) and the recommendations of the Central Ethics Committee in Research of the Autonomous City of Buenos Aires (37), the collection of biological samples intended for future research is contemplated, so that-exclusively in those centers selected to participate in the pathophysiological substudy-a broad consent will be obtained for the use of samples and data in future research on COVID-19.

The study will be conducted in compliance with current national and provincial human subjects research legislation. In addition, all study procedures will comply with the principles of the Declaration of Helsinki and the International Council for Medical Sciences (CIOMS) guidelines.

The protocol will be submitted for evaluation and approval by each of the Research Ethics Committees of the centers invited to participate, or subrogated committees if applicable.

In addition, the protocol will be submitted to the Joint Commission for Health Research in accordance with the provisions of Law 11.044 of the Province of Buenos Aires. It will also be registered in the Health Research Registry (ReNIS) and in the Platform for the Computerized Registry of Health Research of the Autonomous City of Buenos Aires (PRIISA.BA), as required by Resolution 1480/11 of the National Ministry of Health and Resolution 1679/19 of the Ministry of Health of the Autonomous City of Buenos Aires,

respectively. Likewise, it will be registered in each of the jurisdictions where centers are incorporated to the implementation of the protocol, if applicable, in compliance with the provincial regulations in force.

Before proceeding to the data collection, blood sampling and administration of the study medication, the researchers of the center will provide each subject -provided that he/she meets the inclusion criteria and does not present exclusion criteria- with an explanation of the procedures and objectives of the study and will invite him/her to participate. To this end, the subject will be given the study information sheet and will be asked to sign an informed consent form in the presence of a witness, belonging to the center's health care team, but who must be external to the research team. In those institutions where, due to COVID-19 patient isolation rules, it is not possible to take the informed consent form in paper format from the room, the researcher will be accompanied by any member of the care team (but external to the research team) -who will act as a witness to the informed consent process- and will carry with him/her only one original of the consent form. If the patient agrees to participate, he/she will be asked to sign the original consent form provided by the investigator, which will remain in the patient's possession until he/she is allowed to leave or the isolation is completed. If, due to the physical layout of the place of internment of the center, the admission of a person to act as a witness implies a risk for him/her, the witness may participate in the process of taking informed consent virtually, either by telephone or video call. In the centers selected for the pathophysiological substudy, in the presence of the witness, he/she will be asked to give his/her consent to participate in the substudy and to have the blood sample taken. If the center uses a second original of the consent form, it may be signed by the investigator and the witness on the same date but at a later date, and a clarification to this effect will be inserted in the clinical record. In all cases, the research team will exhaust all instances to verify and evidence the patient's signature on the originally signed form, which should be done by means of a digital photograph that will be sent to the intervening witness and to the Executive Committee of the study and whose printout will be kept in the Study's Central Archive.

In each center, the PI will present a sworn statement referring to the respect for the patient's rights and the recording in the clinical history of the consent taking process.

In accordance with the ethical and operational guidelines for the ethical evaluation of research related to COVID-19 issued by the national health authority, it is pertinent to clarify that the present study does not compromise the response to the health emergency established by the pandemic, nor does it unduly interfere in the care of affected persons or in the work of health personnel. Likewise, this research has social value and is relevant because of the potential application of its results to the care of people affected by the disease, and the knowledge sought to be obtained could not be generated by other alternative means or outside the health emergency situation.

The alternatives described in relation to obtaining verbal informed consent are exceptional for the centers and/or cases in which it is not possible to obtain it in writing in the same act. Both the authorization of verbal consent for exceptional cases and the authorization to sign a single informed consent document (copies of which are sent by digital photograph) are part of the risk minimization plan to take extreme measures to avoid contagion and dissemination of the disease within the framework of this research and are subject to the approval of the institutional IRB, depending on the conditions of care and isolation of each center.

Likewise, obtaining verbal consent - in the centers or cases in which it was not possible to obtain it in writing in the same act - is pertinent in the framework of the health emergency, considering that in some cases requiring written consent may imply a risk of contagion, in accordance with the CIOMS guidelines (38), the recommendations of the CCE of the CABA and the document of Ethical Guidance on issues raised by the pandemic of the new coronavirus of the Pan American Health Organization (39).

In those centers in which, for reasons of dynamics and care conditions, the institutional IRB exceptionally authorizes the obtaining of verbal consent, the PI of the center should submit an affidavit to the IRB committing to respect the ethical considerations and the rights of the research subjects, in particular all the considerations referred to the process of informed consent.

3. Definitions

- **Arterial hypertension:** arterial hypertension will be considered to be those cases with blood pressure greater than 140/90 mmHg at admission, and which

the patient reports having a diagnosis of arterial hypertension or being medicated with antihypertensive drugs.

- **Diabetes:** patients who are on anti-diabetic drugs or have a history of diabetes and receive only dietary treatment will be considered diabetic.
- **COPD:** patients receiving chronic obstructive pulmonary disease (COPD) treatment with bronchodilators in chronic form will be considered as COPD carriers.
- **Asthma:** patients with this history will be considered as asthmatic.
- **Cardiovascular disease:** Patients with a history of myocardial infarction, heart failure, percutaneous coronary angioplasty, revascularization surgery (CABG) and/or previous valve replacement will be considered as having cardiovascular disease.
- **Cerebrovascular disease:** Patients with a history of previous stroke (with or without neurological sequelae at the time of study entry) will be considered as carriers of cerebrovascular disease.
- **Obesity**: Patients whose Body Mass Index is ≥30 (estimated according to weight in kilograms divided by height in meters squared) will be considered obese.
- **Respiratory SOFA:** will be measured as a 4-point scale (Figure 4) in which a higher score indicates more impairment. It is estimated by dividing the partial pressure of O_2 in arterial blood (PaO_2) by the Inspired Fraction of O_2. PaO_2 will be estimated from O_2 saturation by pulse oximetry (SpO_2) (25). The score will be calculated from FiO_2 and SpO_2 data reported by the investigators. All SpO_2 measurements will be performed after 5 min of any change in O_2 input so that steady state is reached.

Figure 4: Respiratory SOFA.

	1	2	3	4
PaO_2/FiO_2 (mm Hg)	<400	<300	<220	<100

-Oxygen saturation: O_2 saturation will be used to calculate the difference between the O_2 saturation at admission and the lowest O_2 saturation recorded during hospitalization, breathing room air. This calculation will be performed as the subtraction for each patient and the mean differences between the groups will be compared. In patients in whom, due to their clinical condition, it is not possible to suspend oxygen supply, a saturation of 89% will be attributed. All saturation measurements by pulse oximetry and after 5 minutes of suspending O_2 supply to reach a stable state (40).

- **Quick SOFA:** The quick SOFA (qSOFA) score will be evaluated at admission and according to daily measurements. It contains three variables: arterial hypotension (systolic blood pressure less than or equal to 100 mmHg), tachypnea (respiratory rate equal to or greater than 22 breaths per minute) and altered mental status (Glasgow less than 15 - any degree of mental alteration indicates a Glasgow less than 15). The score is from 0 to 3, more points indicate higher risk (41). For the end point, the qSOFA at admission and the worst recorded during hospitalization will be calculated for each patient and the means will be compared between groups.
- **Requirement for assistance with high-flow oxygen therapy (>40% FIO_2), indication for noninvasive or invasive ventilation:** will be considered as the occurrence of this endpoint in case O_2 treatment is initiated to achieve an inspired fraction greater than 40% (more than 5 liters per minute of O_2 through nasal cannula or face mask), or noninvasive mechanical ventilation is initiated with continuous positive airway pressure (CPAP) or bilevel positive airway pressure (BiPAP) through any adaptive mask, or invasive mechanical ventilation is initiated through orotracheal (or nasotracheal) intubation.
- **Referral to Intensive Care due to clinical needs:** The occurrence of this end point will be considered as the occurrence of this end point in those cases in which the worsening of the patient's clinical conditions makes it necessary for the patient to be admitted to an Intensive Care Unit (ICU), according to the criteria of the treating physician.

- **Invasive mechanical ventilation:** this end point will be considered to occur from the start of mechanical ventilation through orotracheal or nasotracheal intubation.
- **Stroke:** focal loss of a neurological function caused by an ischemic or hemorrhagic event with residual symptoms greater than 24 hours or leading to death.
- **Myocardial infarction:** development of precordial pain, new Q waves and enzyme elevation compatible with the IV definition of infarction.
- **Acute kidney injury:** (or exacerbation of chronic renal failure): 50% increase in initial creatinine levels during evolution.
- **Pulmonary thromboembolism:** suggestive clinical picture (dyspnea, hemodynamic decompensation) confirmed by invasive angiography or angio-tomography.
- **Hospital length of stay:** will be the difference in time in days from admission to discharge from the hospital.
- **ICU length of stay:** will be the difference in days from ICU admission to discharge. In patients where no ICU was required, this will be assigned as 0.
- **In-hospital death:** death from any cause from randomization to discharge or 30 days, whichever comes first, will be evaluated.

4. Potential risks and risk minimization

The randomized, double-blind design minimizes confounding bias. Stratified randomization will be performed to ensure the balanced distribution of two variables of great prognostic weight such as age and diabetes.

The broad selection criteria are intended to include as many patients with uncomplicated disease onset but at risk of developing respiratory complications as possible. This is intended to reduce the risk of selection bias that could also affect the power of the study.

Another source of potential bias is loss to follow-up (attrition); to minimize this, the therapeutic scheme under study was simplified and follow-up was limited to the in-hospital period.

Other biases such as differential assessment or differential reporting will be minimized with the double-blind design.

To evaluate the end points foreseen in each stage of the trial, patients will be randomized and the evaluation of events will be blinded to the assigned treatment, in order to reduce possible selection or measurement biases. By means of randomization and through the estimated sample, it is expected that the distribution between both groups will be balanced, both in terms of population characteristics (sex, history, etc.) and in terms of eventual concomitant treatments. To ensure homogeneous distribution of age and diabetes between the groups, a stratified randomization scheme is used (see "Randomization" section).

The trial does not aim to evaluate the effect of other treatments or interventions; however, it is foreseen in the eCRF the registration of all treatments received by the subjects enrolled in the trial, so that, at the discretion of the Data Safety and Monitoring Board and Executive Committee, the relevant data analyses can be performed to elucidate effects possibly associated with other treatments.

5. Expected results and dissemination of results

The study evaluates a low-cost, single-dose treatment with an excellent safety profile that has immunomodulatory properties in the antiviral response. If effective, it could provide a widely available treatment that would be of great health relevance in the context of the coronavirus pandemic.

The results of the study will be disseminated to the medical community through publication in peer-reviewed biomedical journals and scientific communications.

The results will be published in a global manner, without personal data that could identify the participants, in accordance with current legislation regarding the confidentiality and privacy of research subjects.

6. References

[1]. Disponible en https://coronavirus.jhu.edu/data/mortality, accedido el 2/05/2020.

[2]. Hoffmann M, Kleine-Weber H, Schroeder S, Kruger N, Herrler T, Erichsen S, Schiergens T, Herrler G, Wu NH, Nitsche A, Muller MA, Drosten Ch, Pohlmann S. SARS-CoV-2 Cell Entry Depends on ACE2 and TMPRSS2 and Is Blocked by a Clinically Proven Protease Inhibitor. Cell 2020: 181, 1–10

[3]. Kuba, Y. Imai, S. Rao, H. Gao, F. Guo, B. Guan, Y. Huan, P. Yang, Y. Zhang, W. Deng, L. Bao, B. Zhang, G. Liu, Z. Wang, M. Chappell, Y. Liu, D. Zheng, A. Leibbrandt, T. Wada, A.S. Slutsky, D. Liu, C. Qin, C. Jiang, J.M. Penninger, A crucial role of angiotensin converting enzyme 2 (ACE2) in SARS coronavirus-induced lung injury, Nat. Med. 11 (2005) 875-879.

[4]. M. Vaduganathan, O. Vardeny, T. Michel, J. J.V. McMurray, M. A. Pfeffer, S. D. Solomon, Renin–Angiotensin–Aldosterone System Inhibitors in Patients with Covid-19, The New England Journal of Medicine. (2020). https://doi.org/10.1056/NEJMsr2005760.

[5]. M.L. Sun, J.M. Yang, Y.P. Sun, G.H. Su, Inhibitors of RAS Might Be a Good Choice for the Therapy of COVID-19 Pneumonia, Zhonghua Jie He He Hu Xi Za Zhi. 43(2020):E014. https://doi.org/10.3760/cma.j.issn.1001-0939.2020.0014.

[6] H. Cheng, Y. Wang, G.Q. Wang, Organ-protective Effect of Angiotensin-converting Enzyme 2 and its Effect on the Prognosis of COVID-19, J. Med. Virol. 2020. https://doi.org/10.1002/jmv.25785.

[7]. A.H.J. Danser, M. Epstein, D. Batlle, Renin-Angiotensin System Blockers and the COVID-19 Pandemic: At Present There Is No Evidence to Abandon Renin-Angiotensin System Blockers, Hypertension. 2020. https://doi.org/10.1161/HYPERTENSIONAHA.120.15082.

[8]. Kuba, Y. Imai, S. Rao, H. Gao, F. Guo, B. Guan, Y. Huan, P. Yang, Y. Zhang, W. Deng, L. Bao, B. Zhang, G. Liu, Z. Wang, M. Chappell, Y. Liu, D. Zheng, A. Leibbrandt, T. Wada, A.S. Slutsky, D. Liu, C. Qin, C. Jiang, J.M. Penninger, A crucial role of angiotensin converting enzyme 2 (ACE2) in SARS coronavirus-induced lung injury, Nat. Med. 11 (2005) 875-879.

[9]. M. Ferder, F. Inserra, W. Manucha, L. Ferder, The world pandemic of vitamin D deficiency could possibly be explained by cellular inflammatory response activity induced by the renin-angiotensin system, Am. J. Physiol. Cell. Physiol. 304 (2013) C1027-39.

[10]. D.A. Jolliffe, C.J. Griffiths, A.R. Martineau, Vitamin D in the prevention of acute respiratory infection: systematic review of clinical studies, J. Steroid. Biochem. Mol. Biol. 136 (2013) 321-329. https://doi.org/10.1016/j.jsbmb.2012.11.017.

[11]. J. Charan, J.P. Goyal, D. Saxena, P. Yadav, Vitamin D for prevention of respiratory tract infections: A systematic review and meta-analysis, J. Pharmacol. Pharmacother. 3 (2012) 300-303. https://doi.org/10.4103/0976-500X.103685.

[12]. A.R. Martineau, D.A Jolliffe, L. Greenberg, J.F. Aloia, P. Bergman, G. Dubnov-Raz, S. Esposito, D. Ganmaa, A.A. Ginde, E.C. Goodall, C.C. Grant, W. Janssens, M.E. Jensen, C.P. Kerley, I. Laaksi, S. Manaseki-Holland, D. Mauger, D.R. Murdoch, R. Neale, J.R. Rees, S. Simpson, I. Stelmach, G. Trilok Kumar, M. Urashima, C.A. Camargo, C.J. Griffiths, R.L. Hooper, Vitamin D supplementation to prevent acute respiratory infections: individual participant data meta-analysis, Health Technol. Assess. 23 (2019) 1-44. https://doi.org/10.1136/bmj.i6583.

[13]. E.C. Goodall, A.C. Granados, K. Luinstra, E. Pullenayegum, B.L. Coleman, M. Loeb, M. Smieja, Vitamin D3 and gargling for the prevention of upper respiratory tract infections: a randomized controlled trial, BMC Infect. Dis. 19 (2014) 14:273. https://doi.org/10.1186/1471-2334-14-273.

[14]. D.A. Jolliffe, L. Greenberg, R.L. Hooper, C. Mathyssen, R. Rafiq, R.T. de Jongh, C.A. Camargo, C. J. Griffiths, W. Janssens, A.R. Martineau, Vitamin D to prevent exacerbations of COPD: systematic review and meta-analysis of individual participant data from randomised controlled trials, Thorax 74 (2019) 337–345. http://dx.doi.org/10.1136/thoraxjnl-2018-212092.

[15]. C. Cui, P. Xu, G. Li, Y. Qiao, W. Han, C. Geng, D. Liao, M. Yang, D. Chen, P. Jiang, Vitamin D receptor activation regulates microglia polarization and oxidative stress in spontaneously hypertensive rats and angiotensin II-exposed microglial cells: Role of renin-angiotensin system, Redox Biol. 26 (2019) 101295. https://doi.org/10.1016/j.redox.2019.101295.

[16]. A. Turin, J.J. Bax, D. Doukas, C. Joyce, J.J. Lopez, V. Mathew, G. Pontone, F. Shah, S. Singh, D.J. Wilber, M.G. Rabbat, Interactions Among Vitamin D, Atrial Fibrillation, and the Renin-

Angiotensin-Aldosterone System, Am. J. Cardiol. 122 (2018) 780–784. https://doi.org/10.1016/j.amjcard.2018.05.013.

[17]. D. Carrara, R.M. Bruno, A. Bacca, S. Taddei, E. Duranti, L. Ghiadoni, G. Bernini, Cholecalciferol treatment downregulates renin-angiotensin system and improves endothelial function in essential hypertensive patients with hypovitaminosis D, J. Hypertens. 34 (2016) 2199–2205. https://doi.org/10.1097/HJH.0000000000001072.

[18]. P.S. Leung, The Modulatory Action of Vitamin D on the Renin-Angiotensin System and the Determination of Hepatic Insulin Resistance, Molecules. 24 (2019) pii: E2479. https://doi.org/10.3390/molecules24132479.

[19]. Y.C. Li, G. Qiao, M. Uskokovic, W. Xiang, W. Zheng, J. Kong, Vitamin D: a negative endocrine regulator of the renin-angiotensin system and blood pressure, J. Steroid. Biochem. Mol. Biol. 89-90 (2004) 387-392. https://doi.org/10.1016/j.jsbmb.2004.03.004.

[20]. D. Santoro, D. Caccamo, S. Lucisano, M. Buemi, K. Sebekova, D. Teta, L. De Nicola L, Interplay of vitamin D, erythropoiesis, and the renin-angiotensin system, Biomed. Res. Int. 2015 (2015) 145828. https://doi.org/10.1155/2015/145828.

[21]. J. Xu, J. Yang, J. Chen, Q. Luo, Q. Zhang, H. Zhang, Vitamin D alleviates lipopolysaccharide-induced acute lung injury via regulation of the renin-angiotensin system, Mol. Med. Rep. 16 (2017) 7432–7438. https://doi.org/10.3892/mmr.2017.7546.

[22]. J. Kong, X. Zhu, Y. Shi, T. Liu, Y. Chen, I. Bhan, Q. Zhao, R. Thadhani, Y.C. Li, VDR attenuates acute lung injury by blocking Ang-2-Tie-2 pathway and renin-angiotensin system, Mol. Endocrinol. 27 (2013) 2116-2125. https://doi.org/10.1210/me.2013-1146.

[23]. Y. Shi, T. Liu, L. Yao, Y. Xing, X. Zhao, J. Fu, X. Xue, Chronic vitamin D deficiency induces lung fibrosis through activation of the renin-angiotensin system, Sci. Rep. 7 (2017) 3312. https://doi.org/10.1038/s41598-017-03474-6.

[24]. Grant W, Lahore H, McDonnell S, Baggerly C et. al. Vitamin D Supplementation Could Prevent and Treat Influenza, Coronavirus, and Pneumonia Infections. doi: 10.20944/preprints202003.0235.v1

[25]. Laird E, Kenny RA. Vitamin D deficiency in Ireland –implications for COVID-19. Results from the Irish Longitudinal Study on Ageing (TILDA). https://www.doi.org/10.38018/TildaRe.2020-05

[26]. Kearns MD, Alvarez JA, Tangpricha V. Large, single-dose, oral vitamin D supplementation in adult populations: a systematic review. Endocr Pract. 2014 Apr;20(4):341-51. doi: 10.4158/EP13265.RA.

[27]. Gimenez M, Inserra F, Tajer C, Mariani J, Ferder L, Manucha W. Lungs as Target of COVID-19 Infection: Protective Common Molecular Mechanisms of Vitamin D and Melatonin as a New Potential Synergistic Treatment. Life Sciences, 2020. Aceptado para publicación.

[28]. Pandharipande PP, Shintani AK, Hagerman HE, St Jacques PJ, Rice TW, Sanders NW, Ware LB, Bernard GR, Ely EW. Derivation and validation of Spo2/Fio2 ratio to impute for Pao2/Fio2 ratio

in the respiratory component of the Sequential Organ Failure Assessment score. Crit Care Med. 37 (2009) 1317-21. doi: 10.1097/CCM.0b013e31819cefa9.

[29]. Premaor MO, Scalco R, da Silva MJ, Froehlich PE, Furlanetto TW. The effect of a single dose versus a daily dose of cholecalciferol on the serum 25-hydroxycholecalciferol and parathyroid hormone levels in the elderly with secondary hyperparathyroidism living in a low-income housing unit. J Bone Miner Metab. 26 (2008) 603-8. doi: 10.1007/s00774-008-0858-0.

[30]. Marcinowska-Suchowierska E, Kupisz-Urbanska M, Łukaszkiewicz J, Płudowski,P, Jones G. Vitamin D Toxicity–A Clinical Perspective. Front. Endocrinol. 2018; 9:550.

[31]. Kearns MD, Alvarez JE, Tangpricha V. Large, single-dose, oral vitamin d supplementation in adult populations: a systematic review Endocr Pract. 2014 Apr; 20(4): 341–351.

[32]. The National Heart, Lung, and Blood Institute PETAL Clinical Trials Network . Early High-Dose Vitamin D3 for Critically Ill, Vitamin D–Deficient Patients . N Engl J Med 2019;381:2529-40.

[33]. Rossini M, Gatti D, Viapiana O, Fracassi E, Idolazzi L, Zanoni S, Adami S. Short-term effects on bone turnover markers of a single high dose of oral vitamin D_3. J Clin Endocrinol Metab. 97 (2012) E622-6. doi: 10.1210/jc.2011-2448.

[34]. von Restorff C, Bischoff-Ferrari HA, Theiler R. High-dose oral vitamin D3 supplementation in rheumatology patients with severe vitamin D3 deficiency. Bone. 45 (2009) 747-9. doi: 10.1016/j.bone.2009.06.012.

[35]. Documento técnico Comisión Conjunta de Investigaciones en Salud 001/16. Disponible en: http://www.ms.gba.gov.ar/ssps/investigacion/DocTecnicos/TipoInvestigaciones-Identificacionriesgos.pdf. Accedido el 4/05/2020.

[36]. Orientación ética sobre cuestiones planteadas por la pandemia del nuevo coronavirus (COVID-19). OMS 2020.

[37]. Recomendaciones del CCE referidas al Consentimiento Informado en investigaciones sobre COVID 19. Disponible en: https://www.buenosaires.gob.ar/sites/gcaba/files/recomendaciones_del_cce_referidas_al_consentimiento_informado_en_investigaciones_sobre_covid_19.pdf. Accedido el 4/05/2020.

[38]. Organización Panamericana de la Salud y Consejo de Organizaciones Internacionales de las Ciencias Médica. Pautas éticas internacionales para la investigación relacionada con la salud con seres humanos, Cuarta Edición. Ginebra: Consejo de Organizaciones Internacionales de las Ciencias Médicas (CIOMS); 2016.

[39]. Orientación ética sobre cuestiones clave planteadas por el brote. Washington, DC: OPS; 2016. Disponible en: http://iris.paho.org/xmlui/bitstream/handle/123456789/28485/OPSKBR16002_spa.pdf?sequence=10&ua=1. Accedido el 4/05/2020.

[40]. Gruber P, Kwiatkowski T, Silverman R, Flaster E, Auerbach C. Time to equilibration of oxygen saturation using pulse oximetry. Acad Emerg Med. 2 (1995) 810-5.

[41]. Seymour CW, Liu VX, Iwashyna TJ, Brunkhorst FM, Rea TD, Scherag A, Rubenfeld G, Kahn JM, Shankar-Hari M, Singer M, Deutschman CS, Escobar GJ, Angus DC. Assessment of Clinical Criteria for Sepsis: For the Third International Consensus Definitions for Sepsis and Septic Shock (Sepsis-3). JAMA. 315 (2016) 762-74. doi: 10.1001/jama.2016.0288.

1- Appendix

2-

a- Informed consent form

Cholecalciferol to improve the evolution of patients with COVID-19 (CARED)

Informed consent

INFORMATION SHEET FOR PATIENTS

You have been invited to participate in a clinical research study because you meet the initial requirements for participation. This form contains information about the research study. Please read it carefully and ask questions about anything you do not understand. Your participation in this study is strictly voluntary. If you have any questions about this research, you may ask them at any time. If you agree to participate, you may withdraw from the project at any time without any problems or negative consequences for you. The members of the research team will explain to you what the study is about and answer any questions you may have. Once you understand what the study is about and the extent of your participation, if you agree to participate you will be asked to give your consent. Given the particular characteristics of COVID 19 disease, which implies the need for isolation and distance, your consent will be given verbally in the presence of a witness. Two copies of this document will be signed: the first one will remain in your possession while the other copy of the IC form will be signed by the witness together with the researcher, who will remain at the centre. Whenever possible you will be asked to sign both copies at the appropriate time.

Why is this research being carried out?

The spread of coronavirus infection (SARS-COVID-2) that causes COVID-19 disease can affect anyone. Although there is a significant proportion of people who are infected and do not develop symptoms, a smaller percentage may have respiratory symptoms and so far it is known that respiratory complications are the main cause of poor progression. There are several studies that

showed that treatment with vitamin D could prevent respiratory infections of viral origin, although it is not clear if this happens in the VID-19 infection. The action of the vitamin D could be associated to the modification of the system of defenses of the organism, and this way an extra contribution of this vitamin could contribute to diminish the inflammatory reaction that generates the virus improving the capacity to defend us of the same one. Thus, this research aims to assess whether an extra dose of vitamin D is associated with a lower rate of respiratory complications in a population of patients hospitalized for VID-19.

What will happen if you agree to participate?

If you agree to participate in this study, after obtaining your consent you will be asked some questions about your medical history. If you do not have a blood calcium level test or if you do not know if you have ever had high calcium or risk of having high blood calcium, the professional may ask you to have a blood test to determine your blood calcium level and make sure that you do not have increased calcium values, after which you will be given the study medication. You must take five softgels at the time of admission to the study, one time only. During the first 7 days of hospitalisation your vital signs will be recorded (blood pressure, heart rate, breathing rate and axillary temperature) as well as your O2 saturation measured by a pulse oximeter. All these procedures are part of the usual care of a patient with this pathology. In the same way, all the clinical events that may happen during the hospitalisation will be recorded. These controls will be carried out exclusively while you are in hospital. No controls will be necessary after discharge.

Who is promoting this study?

This study is promoted by the National Ministry of Science, Technology and Innovation, and financed by the National Agency for the Promotion of Science and Technology through the COVID-19 Call for Proposals. It is an independent study, developed by a team of researchers from the Hospital de Alta Complejidad El Cruce, Universidad Nacional Arturo Jauretche, Universidad Maimónides, Hospital Universitario Austral, and Universidad de Cuyo-CONICET. The Principal Investigator of the multicentre study is Dr. Walter Manucha, pharmacologist, independent researcher of CONICET and director of the Laboratory of Experimental, Basic and Translational Pharmacology of the National University of Cuyo.

Where does the study take place?

The research is carried out in a network of care centres in the Province of Buenos Aires, the City of Buenos Aires, the Province of Mendoza and other jurisdictions in Argentina.

What does the study consist of?

El estudio evaluará la evolución respiratoria de adultos con diagnóstico de infección por coronavirus que requieren internación en un centro asistencial. Consta de dos etapas, se espera incluir 200 pacientes en la primera etapa. Si los resultados obtenidos en la primera etapa fueran favorables, se incorporarán 1065 participantes más, hasta completar un total de 1265 pacientes. Para ello se comparará el efecto de una dosis única de vitamina D con una sustancia inactiva farmacológicamente en dos grupos de pacientes, que serán asignados a través de un sistema de asignación por azar, para asegurar que los resultados que se obtengan sean válidos y confiables. Ni los pacientes ni los investigadores de cada centro sabrán si el tratamiento asignado contiene la vitamina o el placebo, solo un Comité independiente del estudio que monitorea los datos y la seguridad podrá acceder a esa información. The study will evaluate the respiratory evolution of adults diagnosed with coronavirus infection who require hospitalisation in a care centre. It consists of two stages; it is expected to include 200 patients in the first stage. If the results obtained in the first stage are favourable, a further 1065 participants will be included, making a total of 1265 patients. For this purpose, the effect of a single dose of vitamin D with a pharmacologically inactive substance will be compared in two groups of patients, who will be assigned through a randomization system, to ensure that the results obtained are valid and reliable. Neither the patients nor the researchers at each centre will know whether the assigned treatment contains the vitamin or the placebo, only an independent Study Committee monitoring the data and safety will be able to access this information.

What are the risks of participating in this research?

The research does not involve risky interventions or invasive procedures. All data to be collected for study purposes are part of routine procedures in a hospital. The only intervention is the administration of 5 softgels which may contain either vitamin D or placebo. The placebo is a substance that does not have any active ingredient, therefore it has no associated risks. Vitamin D

is a nutritional supplement available on the Argentine market and is very safe, in doses lower than those used in this study. Very few adverse effects have been described but with higher doses of vitamin D than those to be used in this study. Some of the effects could be: digestive discomfort, nausea, vomiting, lack of appetite, constipation, weakness. Although there are no reported cases of increased blood calcium levels to risky levels with a single dose of 500,000 units, cases of increased blood calcium have been observed after several months of daily intake of these doses. While there are no reports of a single 500,000 IU dose of vitamin D causing acute kidney injury or failure or kidney stones, patients with symptoms of elevated blood calcium levels or known sarcoidosis should not be included, so if you do not have a blood calcium measurement within the past year and/or if your doctor considers it necessary, he or she will request an analysis before giving you the medication for safety. COVID 19 disease does not yet have a specific treatment. Efforts are being made worldwide to find alternative therapies to improve the course of this disease.

What are the possible benefits of participating in this study?

You will not have any direct benefit from participating; although it is possible that vitamin D may be helpful in reducing the respiratory complications of COVID-19 disease. If proven useful, the impact on the health system could be very relevant as it is a low-cost, safe and available preventive intervention with the potential to treat the disease in a large number of people.

Will it cost anything to participate in the study?

Participation in this research study will not have any cost to you or your health coverage.

What will happen if you decide to withdraw from the study?

If you decide to withdraw from the study, there will be no consequences for the care you are receiving. You should only inform your treating physician or the research team that invited you to participate. You may also be discontinued by decision of the Researcher. If this happens, the reasons will be explained to you and your usual medical care will not be affected.

How will your health information be used?

The information collected about your health will be kept confidential and will only be used for this research. Your data and answers to the questionnaire will be coded using an identification number and will therefore be anonymous. The information may be required by the health authorities (Ministry of Health, Research Ethics Committee; Regulatory Authorities, etc). On occasion your data may be used in other countries and these may not have the same level of protection; however we will ensure that the minimum level of protection guaranteed in this consent form is provided.

In accordance with the Personal Data Protection Act, you have the right to request access to and correction of your personal data. This permission to share your personal health information for this study will be maintained for as long as necessary for the purposes of the study. If you no longer wish to share your personal health information, you may cancel your permission at any time by notifying study personnel and/or the study physician in writing at the address provided on this Informed Consent Form. As stated in Law No. 25,326, you have the right to access your information free of charge and the ability to rectify it. In case you cannot access them, you have the right to make the corresponding report to the National Direction of Personal Data Protection, Control Organ of Law Nº 25.326, (with address in Sarmiento 1185 - 5th floor "p" - C1041AAX - Autonomous City of Buenos Aires - Telephone (011) 4383-8512/3, e-mail: infodnpdp@jus.gov.ar), which is in charge of attending to the complaints and claims that are filed in relation to the breach of the rules on personal data protection.

Who evaluated this research?

This study has been authorized by the Joint Commission of Health Research of the Province of Buenos Aires and by the Committee of Ethics in Research of the Ministry of Health of the Province of Mendoza. In addition, it was evaluated and approved by the Ethics Committee of the centre: [insert name of the centre], [address of the centre's CEI] whose president/coordinator is [name of the authority of the CEI].

Studio insurance

By signing this informed consent you do not waive the rights you have under the Civil Code and Argentine laws on civil liability for damages.

This study has an insurance that will cover the expenses that may be incurred for the realization of the study guaranteeing the coverage of the risks or potential damages that could be derived from the participation in the study (Policy nº 8176793, NOBLE COMPAÑÍA DE SEGUROS S.A.).

CONTACT INFORMATION

If you have any questions regarding your rights as a research participant, you may contact the Committee [name of the Institutional Ethics Committee, name of the Committee's referee, address, telephone number and e-mail address, Committee hours of operation, if applicable].

If, in the course of the research, doubts or questions arise about your participation in this study, you may contact [Name and surname of the centre's PI] by telephone: [provide a telephone number and if possible a mobile phone number for contact] and/or e-mail: [provide e-mail address].

INFORMED CONSENT

I agree to voluntarily participate in this research. I have read and understand the contents of the Study Participant Information Sheet.

I have been adequately informed about the objectives, risks and benefits of the study. The characteristics of the research have been clearly explained to me and all my questions have been answered. I have been informed that I can ask questions about the project at any time.

I understand that my participation is free and voluntary, that there is no cost or payment to me or my coverage. I have been told that I can withdraw from the study at any time without harm or consequence of any kind.

I agree to follow the instructions given to me by the research team.

I know that the information I provide during this research is strictly confidential and will not be used for any other purpose without my consent.

I understand that I will be given an original of this consent form, and that I may request information about the results of this study when the research has been completed.

I agree to participate in this research

YES □ NO □

I agree that my clinical data may be used for scientific publications, provided that my personal data are concealed

YES □ NO □

Name and Surname Patient or Legal Representative	Signature	DNI	Date
Name and Surname Witness	Signature	DNI Witness	Date
Name and Surname Researcher	Signature	DNI Researcher	Date

If the circumstances of isolation so require, a single copy will be signed, of which the research team will take a photograph in duplicate to prevent paper-based transmission of the COVID-19 pandemic.

b- Informed consent form for the physiopathological sub-study.

PHYSIOPATHOLOGICAL SUB-STUDY

MULTICENTRE CONTROLLED TRIAL WITH HIGH DOSE VITAMIN D VERSUS PLACEBO TO PREVENT EVOLUTIONARY COMPLICATIONS IN COVID-19 INFECTED PATIENTS

INFORMATION SHEET FOR PATIENTS

Because you agreed to participate in the "High-dose vitamin D versus placebo controlled trial to prevent evolutionary complications in patients infected with COVID-19 (CARED)", we invite you to participate in this "physiopathological sub-study", which aims to evaluate in blood samples some markers related to the inflammatory response in patients affected by COVID-1 and to store a part of them for future biomedical research studies. Your participation in this sub-study is strictly voluntary and confidential. If you have any questions about this project, you can ask them at any time. You may also withdraw from the sub-study at any time without prejudice in any way.

If you agree to participate, 10-15 ml of blood will be drawn after you sign this document and a second 10 ml sample will be drawn between the 3rd and 7th day after you receive your study medication, or at the time of discharge (if this is before 72 hours after taking your study medication). These samples, after being processed under special conditions, will be stored in freezer at -80 C, to be analyzed in the future. No additional visit or extraction is necessary.

What are the risks of participating in this research?

The risks are the same as those you may experience when having blood drawn for checks during your stay. For most people, needlesticks for blood draws do not cause serious problems. However, they can cause bruising, discomfort, nerve damage, infection and/or pain where the needle or frame is inserted. If you feel that you are going to faint, inform your doctor or the study staff immediately.

What are the possible benefits of participating in this study?

You will not have any direct benefit from participating in this biomedical research. However, this research may contribute to our understanding of SARS-CoV 2 and the effects of vitamin D in these cases.

Will it cost anything to participate in the study?

ou will not be paid for participating in this sub-study. Costs related to the collection, transport and storage of samples and their analysis will be covered by the research.

What will happen if you decide to withdraw from the study?

If you decide to withdraw from the study, there will be no consequences for the care you are receiving. You should only inform your treating physician or the research team that invited you to participate, so that they can destroy the stored samples.

Do I have to participate?

It is up to you to decide whether or not to participate in this sub-study for biomedical research. You can refuse to donate a sample at any time, without causing problems or losing the benefits to which you are entitled anyway. Even if you decide not to donate a sample, you can still be part of the main study.

What rights do I have to see the results of biomedical research?

The results of the biomedical study will not be made available to you, as they may be inconclusive or not properly interpreted. If any of these results have a clear and direct medical consequence for you, the information will be shared with you through the study research team or another professional who can properly interpret the information. Test information will not be part of your medical record and will not be provided to your health insurance or employer. In addition, the consent form for the main study provides more detailed information about the handling of your study data and samples. Your samples and medical information will be maintained with the same level of privacy described above for the main study.

What rights do I have with regard to the results of biomedical research?

Any information derived directly or indirectly from this biomedical research, as well as any patents, medicines or biological products developed directly or indirectly as a result of this research, are the exclusive property of the sponsor. However, by signing this form and donating a blood sample for research, you do not waive any rights you would otherwise have as a participant in this research. This is without prejudice to the legal effects that Argentinean law gives to the research subject with respect to the ownership of his/her samples and the right to withdraw consent. Please refer to the "How will your health information be used" section of the Informed Consent Form for the main study.

Beyond the current study, any new research study using your coded samples will be reviewed by the Research Ethics Committee (REC) involved in the evaluation of the main study.

CONTACT INFORMATION

f, during the course of the research, doubts or questions arise about your participation in this study, you can contact [Name and surname of the centre's IP] by telephone: [provide line telephone number and if possible a contact mobile phone] or by e-mail [email address].

This research work has been evaluated by the Research Ethics Committee [indicate data of the IRB involved], registered in the Provincial Registry of Research Ethics Committees, under the Central Research Ethics Committee - Ministry of Health of the Province of Buenos Aires with date [insert accreditation/re-accreditation date, according to IRB operating rules], registered in: If you have any questions regarding your rights as a research participant, you may contact the Research Ethics Committee [insert details of the IRB that took part and the contact person, address, telephone numbers and e-mail address, and opening hours, if applicable].

In accordance with the Personal Data Protection Act, you have the right to request access to and correction of your personal data. This permission to share your personal health information for this study will be maintained for as long as necessary for the purposes of the study. If you no longer wish to share your personal health information, you may cancel your permission at any time by notifying the study staff and/or the study physician in writing at the address listed on this Informed Consent Form. As stated in Law No. 25,326, you have the right to access your information free of charge and the ability to rectify it. In case you are not able to access them, you have the right to file the corresponding complaint with the National Data Protection Office. The National Personal Data Protection Office, Control Body of Law No 25,326, (with address at Sarmiento 1185 - 5th floor "p" - C1041AAX - Autonomous City of Buenos Aires - Telephone (011) 4383-8512/3, e-mail: infodnpdp@jus.gov.ar), is in charge of attending to the complaints and claims filed in relation to the breach of the rules on personal data protection.

INFORMED CONSENT TO PARTICIPATE IN THE PHYSIOPATHOLOGICAL SUB-STUDY

I agree to voluntarily participate in this physiopathological sub study for biomedical research.

I have read and understand the contents of the Information Sheet for participants in the physiopathological sub-study of the main trial. I have been adequately informed about the objectives, risks, and benefits of the sub-study. The characteristics of the research have been clearly explained to me and all my questions have been answered.

I have been informed that I can ask questions about the study at any time.

I understand that my participation is free and voluntary, that there is no cost or payment to me or my coverage. I have been told that I can withdraw from the study at any time without harm or consequence.

I agree to follow the instructions given to me by the research team.

I know that the information I provide during this research is strictly confidential and will not be used for any other purpose without my consent.

I understand that I will be given an original of this consent form.

I agree to participate in this pathophysiological sub-study.

YES □ NO □

I agree to have a blood sample taken from me for storage at a local biological sample bank for analysis in future scientific research, provided that my personal details are concealed.

YES □ NO □

Name and Surname Patient (or Legal Representative)	Signature	DNI Patient	Date
First and Last Name Witness	Signature	DNI Witness	Date
Name and Surname Researcher	Signature	DNI Researcher	Date

Confidencial
