# Supplementary material for: High-dose vitamin D versus placebo to prevent complications in COVID-19 patients: Multicentre randomized controlled clinical trial
Source: PLoS One. 2022 May 27;17(5):e0267918. doi: 10.1371/journal.pone.0267918 (PMC9140264; doi:10.1371/journal.pone.0267918)
Supplement: S2 File — (PDF) [file pone.0267918.s003.pdf]

Guide for FiO2 estimation among participants with oxygen supplementation in whom a temporary interruption of the supplementation is judged inappropriate.

Administration device	FiO_2 (Liters)
**Nasal canulae**	0.24-0.28 (Max 5 lts)
**Venturi Mask**	0.24 (3 lts)
	0.35 (8 lts)
	0.40 (10 lts)
	0.50 (12 lts)
**Reservoir mask**	0.55 (6 lts)
	0.60 (7 lts)
	0.70 (8 lts)
	0.80 (9 lts)
	0.90-0.99 (10-15 lts)
