# Supplementary material for: High-dose vitamin D versus placebo to prevent complications in COVID-19 patients: Multicentre randomized controlled clinical trial
Source: PLoS One. 2022 May 27;17(5):e0267918. doi: 10.1371/journal.pone.0267918 (PMC9140264; doi:10.1371/journal.pone.0267918)
Supplement: S3 File — (PDF) [file pone.0267918.s004.pdf]

Supporting information file 2

Sites, Principal investigators and number of recruited participants in the The CholecAlcifeRol to improvE the outcomes of patients with COVID-19 (CARED) trial.

Site. Principal investigator, (n):

Hospital de Infecciosas Francisco Javier Muñiz, Ciudad Autónoma de Buenos Aires, Argentina. Milagro Sanchez Cunto (n=39).

Hospital General de Agudos Dr. Enrique Tornú, Ciudad Autónoma de Buenos Aires, Argentina. Diego Brosio (n=32).

Clínica Santa Isabel, Ciudad Autónoma de Buenos Aires, Argentina. Fernando Ross (=30).

Hospital General de Agudos Dr. Cosme Argerich, Ciudad Autónoma de Buenos Aires, Argentina. Marcelo Zylberman (n=20).

Hospital General de Agudos Dr. Teodoro Álvarez, Ciudad Autónoma de Buenos Aires, Argentina. Daniel Emilio López (n=18).

Hospital General de Agudos Dr. Juan A. Fernández, Ciudad Autónoma de Buenos Aires, Argentina. Cecilia Luna Hisano (n=13).

Hospital de Alta Complejidad en Red El Cruce - Néstor Kirchner, Florencio Varela, Buenos Aires, Argentina. Sebastián Maristany Batisda (n=13).

Hospital General de Agudos Parmenio Piñero, Ciudad Autónoma de Buenos Aires, Argentina. Gabriela Pace (n=11).

Hospital Luis Lagomaggiore, Mendoza, Mendoza, Argentina. Adrián Salvatore (n=8).

Sanatorio Güemes, Ciudad Autónoma de Buenos Aires, Argentina. Jimena Fernanda Hogrefe (n=6).

Hospital Regional Antonio J. Scaravelli, Tunuyán, Mendoza, Argentina. Marcela Turela (n=6).

Sanatorio Allende, Sede Cerro, Córdoba, Córdoba, Argentina. Andrés Gaido (n=5).

Hospital Zonal General de Agudos "Dr. Isidoro Iriarte, Quilmes, Buenos Aires, Argentina. Beatriz Rodera (n=5).

Hospital Interzonal Especializado en Agudos y Crónicos. Neuropsiquiátrico Dr. Alejandro Korn, Melchor Romero, Buenos Aires, Argentina. Elizabeth Banega (n=4).

Sanatorio Allende Nueva Córdoba, Córdoba, Córdoba, Argentina. María Eugenia Iglesias (n=4).

Hospital Modular de Florencio Varela, Florencio Varela, Buenos Aires, Argentina.
Mariela Rzepeski (n=3).

Hospital El Carmen, Godoy Cruz, Mendoza, Argentina. Juan Manuel Gomez Portillo (n=1).
