# Supplementary material for: High-dose vitamin D versus placebo to prevent complications in COVID-19 patients: Multicentre randomized controlled clinical trial
Source: PLoS One. 2022 May 27;17(5):e0267918. doi: 10.1371/journal.pone.0267918 (PMC9140264; doi:10.1371/journal.pone.0267918)
Supplement: Attachment — Submitted filename: Response to Reviewers - R2.docx [file pone.0267918.s007.docx]

**Response to Reviewers – R2**

**Journal Requirements:**

Please review your reference list to ensure that it is complete and correct. If you have cited papers that have been retracted, please include the rationale for doing so in the manuscript text, or remove these references and replace them with relevant current references. Any changes to the reference list should be mentioned in the rebuttal letter that accompanies your revised manuscript. If you need to cite a retracted article, indicate the article’s retracted status in the References list and also include a citation and full reference for the retraction notice.

1- **Response:** We reviewed the reference list and searched for retracted articles at: The Retraction Watch Database [Internet]. New York: The Center for Scientific Integrity. 2018. ISSN: 2692-465X.. Available from: <http://retractiondatabase.org/>. (accessed on April 3^rd^ 2022). We found no retracted articles in the reference list.

**Additional Editor Comments:**

**Comment #1:** Methods. The definition of adverse event and/or serious adverse event is missing. Death is considered an adverse event?

**Response #1:** Thank you for the comment. We added the sentence “Serious adverse events were defined as any occurrence in a participant that caused death, was life-threatening, prolonged the hospitalization, caused significant or persistent disability and/or was judged by investigators to represent a significant risk for participant.”, in “Methods”. Death was considered and reported as a Clinical Outcome, not as serious adverse event (i.e. a participant that have had a septic shock that have led to death, has the infectious event reported as a serious adverse event and death as a clinical outcome).

**Comment #2:** Results, adverse events. You are reporting in the text 45 adverse events (or serious adverse events? unclear). However, Table 3 reported only 4 events.

**Response #2:** Thank you very much for this comment. We added the word “serious” to all reference to adverse events to avoid confusion. Please, note that Table 3 reports both the number (and percentage) of participants with at least one serious adverse event and the number of events across systems (we added the neurological serious adverse events). The sum of serious adverse events across systems is 45. Please, let us know if there is need for further clarification.

**Comment #3:** Table 2. This table refer to serious adverse events, correct? Please correct the caption. Furthermore, I suggest to invert the order of the columns (VitD before placebo, as in Table 1 and 2).

**Response #3:** Thank you for the comment. Table 3 refers to serious adverse events. We corrected the caption. Also, we inverted the order of columns.

**Reviewers' comments:**

**Reviewer #1**

**Comment #1:** Globally the authors have addressed the comments and the study is of interest.

but the rSOFA definition remains problematic: either authors have not understood the question … or they do not want to respond. The request was to please provide the ranges of SpO2 and FiO2 used to attribute 0 to 4 points of r-score. The answer is the classical P/FO2 ratios ? “Values of ratios SpO2/FiO2 for rSOFA calculations were as follows: >=400, rSOFA 0; <400 and >=300, rSOFA 1; <… » is an inappropriate answer -

Please profile the SpO2 values that were used to attribute rSOFA scores of 0,… 4.

In my ICU: an SpO2 of 98-100% under 2L/min O2 results would be graded as “1”, etc. (please provide the FiO2 from 2-3-4-5 l/mi O2)

**Response #1:** Thank you for the comment. Definitely, we did not understood the question. For baseline SpO2 was measured breathing room air and the FiO2 was considered 0.21. For follow-up measures among patients with oxygen supplementation the guide provided to investigators was the following table:

| **Administration device** | **FiO_2_ (Liters)** |
| --- | --- |
| **Nasal canulae** | 0.24-0.28 (Max 5 lts) |
| **Venturi Mask** | 0.24 (3 lts) |
|  | 0.35 (8 lts) |
|  | 0.40 (10 lts) |
|  | 0.50 (12 lts) |
| **Reservoir mask** | 0.55 (6 lts) |
|  | 0.60 (7 lts) |
|  | 0.70 (8 lts) |
|  | 0.80 (9 lts) |
|  | 0.90-0.99 (10-15 lts) |

We added the following sentence in “Outcomes and follow-up”: “The rSOFA score was calculated with participant breathing room air, however, for participants with oxygen supplementation requirement and that treating physician judged not posible to temporary suspend, a guide for FiO2 estimation was provided to investigators (S2 File).”. Also, the table is inclued as Supplemental file, now S2 file.

**Reviewer #2**

**Comment #1:** The authors added, “Castor was used for randomization and data collection” to explain how the randomization and treatment allocation was done. However, this is not clear for readers at all. General (international) readers do not know what Castor was.

**Response #1:** Thank you for this comment. We modified the sentence, in the revised version is “Castor®, and electronic data capture plataform that has online randomization capability, was used for randomization and data collection (<https://www.castoredc.com)>.”.

**Comment #2:** If any, please provide the rationale that the authors added a threshold of age >45 as a risk for disease progressing. If there is not, and the threshold was merely set arbitrarily, that would be fine but should be mentioned in the manuscript as such.

**Response #2:** Thank for the comment. We intended to include a population at risk of respiratory worsening during the hospitalization for COVID-19 (the study initiated in a moment of the pandemic when patients were hospilized with a broad spectrum of disease severity, including mild disease). We added the sentence “Age 45 or older was selected as inclusion criterion as an intent to ensure a baseline risk of respiratory worsening that allow to detect a therapeutic effect of the treatment, and to preserve the power of the study.”, in the paragraph describing the inclusion criteria.

**Comment #3:** The authors replied that there were no stroke, myocardial infarction, and pulmonary embolism events. However, this was not reported in the manuscript. Please be noted that all outcomes planned to be measured in the protocol should be reported even if there were no events. “No events” itself is an important finding.

**Response #3:** Thank you for the comment. We added the information in the Table 2.

**Comment #4:** I would suggest the authors provide the result adjusting for an imbalanced variable (asthma or COPD) in the supplementary materials to support the main finding as additional exploratory analysis.

**Response #4:** Thank you for the comment. We added the results of the adjusted analysis in the “Sensitivity analysis” sub-section of “Results”, with the following sentence: “The results of a post-hoc analysis adjusting for the imbalance in the distribution of COPD or asthma between groups, were similar to main results (OR 0.99; 95% CI 0.69 to 1.41; p=0.950).”

**Reviewer #3**

**Comment #1:** For the main analysis of the primary and secondary endpoints (Table 2), please report the sample statistic for the between-group difference in medians, rather than the mean of the smoothed bootstrap distribution; the bootstrapping procedure is necessary only to quantify uncertainty in/estimate a confidence interval for the point estimate from the sample.

**Response #1:** Thank you very much for this comment. We modified in the text and in table 2.

**Comment #2:** I still have some questions about missing data and the primary outcome definition. Was the rSOFA measured daily for all trial participants on all days from admission through discharge? Or were there some participants for whom one or more daily measurements were missing? I ask because--while the outcome definition (which focuses on the change from baseline to highest *recorded* post-baseline rSOFA score) does somewhat skirt the issue of missing data---if any individuals were missing rSOFA measurements for one or more post-baseline days, the recorded change potentially underestimates the true change. And if missing data patterns systematically differ between the two study arms, there could be bias as a result. If there are missed/missing measurements in the data, please comment on this missing data/the missing data patterns as well as any implications for your analysis.

**Response #2:** We had no missing data for rSOFA, all participants had their SpO2 and FiO2 measured from baseline to hospital discharge, the death or seventh day from study entry, whichever came first. During the course of the study great emphasis and efforts were devoted to maintain the missingness at minimum.

**Comment #3:** Grammatical/Typographical Suggestions: Page 5 (revised version): the last sentence of the first paragraph ("Vitamin D reduce pro-inflammatory cytokines, increase those with anti-inflammatory actions...") should be split in two ("these actions could potentially improve clinical outcomes of patients with COVID-19 pneumonia" should be its own sentence) and "reduce"/"increase"/"upregulate" should all be singular ("reduces"/"increases", "upregulates").

**Response #3:** Thank you for this comment. We splited the sentence and corrected the verbs to singular.

**Comment #4:** Page 5 (revised version): "the evidence supporting the role of vitamin D supplementation to treat patients with COVID-19 remain inconclusive..." should use "remains"

**Response #4:** Thank you for the comment. We corrected.

**Comment #5:** Page 8 (revised version): the clause "since was recognized as risk factor after the study begun" is missing an object and should likely read "since it was recognized as a risk factor after the study began".

**Response #5:** Thank you for the comment. We corrected.

**Comment #6:** Page 15 (revised version): "theses percentages for participants in the placebo group were" contains a typo

**Response #6:** Thank you for the comment. We corrected to “these”.

**Comment #7:** Page 20 (revised version): the sentence "Although results of vitamin D3 supplementation for treatment of patients with COVID-19 could be teoretically modified by the serum vitamin D status, with defficient populations" contains two typos

**Response #7:** Thank you for this comment. We corrected and re-formulated the sentence to “Although results of vitamin D3 supplementation for treatment of patients with COVID-19 could be theoretically modified by the serum vitamin D status, with deficient populations obtaining the most benefits, this remains speculative”.

**Comment #8:** Page 21 (revised version): "the study was underpower to detect differences between groups on clinically important events" should read "the study was underpowered"

**Response #8:** Thank you for the comment. We corrected.

**Comment #9:** Page 21 (revised version): the sentence "the measured serum 25-OH VitD levels were sufficient wether different results" contains a typo and is also incomplete.

**Response #9:** Thank for this observation. We corrected the typo and re-formulated the sentence to “In the present study, the measured serum 25-OH VitD levels among the participants with blood samples were sufficient, whether different results would be obtained among a vitamin D deficient population remains to be determined.”

**Comment #10:** Page 21 (revised version): the sentence "The SpO2/FiO2 ratio used as primary outcome have been validated as surrogate of PO2/FiO2, although validation studies did not included pacients with COVID-19, our results were consistent for several measures of respiratory worsening" contains a typo ("pacients"), should be broken into two or more sentences, and should be checked for verb tense and subject/verb agreement

**Response #10:** Thank for this observation. We re-formulated the sentence y two sentences and changed the second part as following to clarify the message: “The SpO2/FiO2 ratio used as primary outcome have been validated as surrogate of PO2/FiO2. Although validation studies of SpO2/FiO2 ratio did not included patients with COVID-19, the absence of effects on other measures of respiratory worsening besides rSOFA, gives reassurance to study results [24,25,38].”

**Reviewer #4**

**Comment #1:** I thank the authors for considerung my comments. I would appreciate it if the results were supplemented by the WMW odds.

**Response #1:** Thank you for the comment. We added the sentence “For primary outcome, the Wilcoxon-Mann-Whitney odds (WMWOdds) with the corresponding 95% CIs was computed (28).” In the “Statistical analysis” section; and we added the WMWOdds for the primary outcome in “Results”. Also, we added the cite of Divine GW DOI: 10.1080/00031305.2017.1305291 (reference #28).
